# S2k Guideline: Hygienic requirements for patient beds, bed linen, bed accessories and personal protection when handling beds

**DOI:** 10.3205/dgkh000656

**Published:** 2026-06-18

**Authors:** Axel Kramer, Julia Seifert, Bernd Gruber, Marianne Abele-Horn, Mardjan Arvand, Alexander Blacky, Michael Buerke, Iris Chaberny, Maria Deja, Steffen Engelhart, Dieter Eschberger, Anja Gerhardts, Achim Hedtmann, Julia Heider, Christian Jäkel, Peter Kalbe, Horst Luckhaupt, Wolfgang Müller, Alexander Novotny, Cihan Papan, Hansjürgen Piechota, Frank-Albert Pitten, Veronika Reinecke, Simone Scheithauer, Dieter Schilling, Walter Schulz-Schaeffer, Ulrich Sunderdiek

**Affiliations:** 1 German Society for General and Hospital Hygiene, Berlin, Germany; 2 German Trauma Society, Berlin, Germany; 3 German Nursing Council, Berlin, Germany; 4 Paul Ehrlich Society for Infection Therapy, Munich, Germany; 5 Robert Koch Institute, Department Infectious Diseases, Unit Hospital Hygiene, Infection Prevention and Control, Berlin, Germany; 6 Austrian Society for Hospital Hygiene, Vienna, Austria; 7 German Cardiac Society, Düsseldorf, Germany; 8 German Society for Hygiene and Microbiology, Münster, Germany; 9 German Society of Anaesthesiology and Intensive Care Medicine, Munich, Germany; 10 Society of Hygiene, Environmental and Public Health Sciences, Müllheim, Germany; 11 Vienna Regional Office of the Austrian Workers’ Compensation Insurance, Vienna, Austria; 12 Hohenstein Institute for Textile Innovation gGmbH (HIT), Bönningheim, Germany; 13 Professional Association of Orthopaedic and Trauma Specialists (BVOU), German Society for Orthopaedics and Trauma, Berlin, Germany; 14 German Society for Oral, Maxillofacial and Facial Surgery, Hofheim am Taunus, Germany; 15 Dr. Jäkel, Medical Law, Pharmaceuticals Law, Medical Devices Law, Luebben, Germany; 16 Professional Association of German Surgery, Berlin, Germany; 17 German Society of Oto-Rhino-Laryngology, Head and Neck Surgery, Bonn, Germany; 18 Former Head of the Office of the Association of the Scientific Medical Societies in Germany; 19 German Society for Surgery, Berlin, Germany; 20 German Society for Pediatric Infectious Diseases, Berlin, Germany; 21 German Society for Urology, Düsseldorf, Germany; 22 German-speaking Interest Group of Experts for Infection Prevention and Consultants for Hospital Hygiene, Zurich, Switzerland; 23 Department of Infection Control and Infectious Diseases, University Medical Center Göttingen (UMG), Georg-August University Göttingen, Göttingen, Germany; 24 German Society for Digestive and Metabolic Diseases, Berlin, Germany; 25 Department of Neuropathology, Medical Faculty of the Saarland University, Homburg/Saar, Germany; 26 German X-ray Society and German Society for Interventional Radiology and Minimally Invasive Therapy, Berlin, Germany

**Keywords:** patient bed, hygienic requirement, ergonomic requirement, patient comfort, reprocessing, disinfection, chemothermal disinfection-washing procedures, final disinfection, reprocessing control, personal protection, bed linen, bed inserts, pillows, neck pillows, blanket core, encasing, barrier cover, changing, nosokomiale Infektion, MRSA, VRE, P. aeruginosa, Acinetobacter, K. pneumoniae; C. difficile; Shigella; C. auris; Norovirus

## Abstract

Hospitalized patients are often more susceptible to infection than healthy people due to their illness, the presence of devices and their reduced immune defenses. At the same time, potentially pathogenic pathogens, which are often characterized by antibiotic resistance, are released into the area close to the patient, including the bed. To prevent hospital beds from becoming a source of nosocomial infections, bed linen and hospital beds, including encasings or pillows and comforters, must be disinfected before reoccupation, in contrast to hotel beds.

The guideline outlines the hygienic and ergonomic requirements for hospital beds, ensuring bed hygiene during the patient's stay and the principles of bed reprocessing, including organization, quality assurance and staff protection, in 40 recommendations.

## Abstract (de)

Krankenhauspatienten besitzen durch ihre Erkrankung, durch u.U. vorhandene Devices und auf Grund herabgesetzter Immunabwehr häufig eine höhere Infektionsanfälligkeit als Gesunde. Zugleich werden in den patientennahen Bereich einschließlich des Betts potenziell pathogene Erreger freigesetzt, die häufig durch Antibiotikaresistenzen gekennzeichnet sind. Um zu verhindern, dass Krankenhausbetten nicht zur Quelle von nosokomialen Infektionen werden, müssen Bettwäsche und Krankenhausbetten einschließlich Encasings bzw. Kissen und Bettdeckenkern im Gegensatz zum Hotelbett vor der Neubelegung situationsgerecht desinfizierend aufbereitet werden.

In der Leitlinie werden die hygienischen und ergonomischen Anforderungen an Krankenhausbetten, die Gewährleistung der Bettenhygiene während des Patientenaufenthalts sowie die Grundsätze der Bettenaufbereitung einschließlich der Organisation, der Qualitätssicherung und des Personalschutzes in 40 Empfehlungen begründet.

 Republication of: Kramer A, Seifert J, Gruber B, Abele-Horn M, Arvand M, Blacky A, Buerke M, Chaberny I, Deja M, Engelhart S, Eschberger D, Gerhardts A, Hedtmann A, Heider J, Jäkel C, Kalbe P, Luckhaupt H, Müller W, Novotny A, Papan C, Piechota H, Pitten FA, Reinecke V, Scheithauer S, Schilling D, Schulz-Schaeffer W, Sunderdiek U. S2k Guideline: Hygienic requirements for patient beds, bed linen, bed accessories and personal protection when handling beds. GMS Hyg Infect Control. 2025;20:Doc20. DOI: 10.3205/dgkh000549

## Recommendations and justification

### 1. Hygienic and ergonomic requirements for patient beds

**1. Recommendation**

*In hospitals and rehabilitation facilities, beds should be used that satisfy the comfort of patients regarding nursing care and well-being and should be adaptable to the patient's situation through various accessories.*

**Recommendation degree:** ↑↑

**Strength of consensus:** >95%

Care beds and hospital beds are identical in terms of their features. However, the different names are an indication of the cost bearer. For example, a doctor may prescribe a hospital bed if the patient has a disability and such a bed makes everyday life easier or is medically necessary. The costs are then covered by the health insurance company.

Care beds are designed for long-term nursing care. The long-term care insurance fund covers the costs if a nursing-care level is recognized and the care bed either facilitates care, alleviates the patient's complaints or helps them to be more independent [1]. 

The height of the head and foot ends of the classic care bed can be adjusted. It usually has lockable castors so that it can be moved when the brake is released. This is especially important for bedridden patients, as it is the only way to change rooms and perspectives.

**2. Recommendation**

*The bed should be risk-adapted to ensure dimensional stability, pressure relief and elasticity, and support the recovery process.*

**Recommendation degree:** ↑

**Strength of consensus: **>95%

Depending on the patient clientele, state of health and body weight of the patient, there are different requirements for the comfort and stability of the bed, which must be clarified at the latest upon admission, because they are not always met by the classic hospital bed. For example, pressure-relieving mattresses (anti-decubitus mattresses) can be used if the patient is expected to be bedridden for a long time and is at risk of developing pressure sores [2], [3]. After spinal surgery, the mattress and slatted frame should be selected in such a way that the spine retains its upright shape when lying down as it does when standing upright (standing bed). For overweight patients, beds with special stability and height adjustability may be required (heavy-duty bed). For patients who can easily fall out of bed, e.g., those with dementia, the care bed should protect them from falling out. If a bed rail is not the right solution, a very low bed can be considered (low-height bed). With a side-support bed, the angle of inclination of the long side of the bed can be adjusted by up to 15 degrees. This can make it easier to wash in bed and prevent pressure sores. The mattress is slotted so that it can be folded. For people with disabilities, special beds are required, e.g., with increased load-bearing capacity and a wider mattress base, motorized height adjustment, motorized adjustment of the mattress base, sitting and stand-up function if necessary and the option of equipping the bed with the required accessories.

Ideally, it should be clarified before hospitalization whether a special bed is required. For this purpose, beds are either kept in the facility or leased (via a hotline, for instance). 

Dimensional stability primarily concerns the properties of the mattress and its spring base. According to DIN 13014 [4], a good quality mattress should have a density of at least 40. A density of 45 to 55 is considered optimal.

Pressure relief is particularly important for bedridden patients. Mattresses with point-elastic core materials react to point loads on the body and allow it to sink in more where the pressure is higher. Mattresses with a pocket spring, latex, visco-foam or gel-foam core offer particularly high point elasticity. By dividing the core into zones, the point elasticity of the mattress is further enhanced. The support areas for the heavier parts of the body are designed, for example, with holes in the core or the use of a softer material so that better sinking into the mattress surface is possible. The pressure-relieving effect of a mattress is further improved by a suitable bed base. For example, there are sprung wooden frames or point-elastic box springs with a multi-zone division, which are particularly flexible in the shoulder or hip/buttock areas and make it easier to sink deeper into the mattress for greater pressure relief [5].

It is advantageous if the covers of mattresses are designed to be elastic, for example due to elastane in the fabric; these are called stretch covers. Elasticity is also important for fitted sheets. An elastic band is usually sewn around the perimeter of the sheet, allowing it to be pulled comfortably over the mattress. It is particularly advantageous if the fitted sheet is made of an elastic textile, so that the fabric still gives when the sheet is pulled up [5].

The neck can be relieved with special neck support pillows. They usually have a special shape and are equipped with a core of pressure-relieving material. Their shape allows the sensitive neck area to be positioned in ergonomically favorably, and the pressure-relieving core ensures that the pillow surface sinks in well. This prevents tension and pain in the neck [5].

**3. Recommendation**

*The patient's thermal comfort should be ensured by water-vapor-permeable materials, comfortable bed linen, bed inserts, pillows and comforter cores.*

**Recommendation degree:** ↑↑

**Strength of consensus: **>95%

The bed furnishings are essential for restful sleep. The bedding – especially the mattress, e.g. encasings – should not provide any additional cause for sweating. Sweating in bed creates an unpleasant feeling, impairs sleep, can promote colds and lead to painful muscular tension.

Good moisture regulation is achieved when the body is kept dry despite sweating. The body moisture is absorbed by the sheet or mattress and the bed linen or comforter and transported from there to the other side of the equipment component (-called moisture wicking). The moisture can evaporate there. Cotton is characterized by especially high moisture absorption; however, moisture is not wicked away, which leads to a damp, cool bed climate with unpleasant consequences in the event of heavy perspiration. Microfiber and silk are characterized by good moisture management. Both absorb moisture well and do not store it, but release it again quickly. Functional materials use the principle of wicking. In bedding, moisture is drawn from the inside of the material to the outside. The moisture should be distributed as quickly as possible over as large an area as possible to achieve the fastest possible evaporation. Mechanical solutions are mainly used to provide the bedding with this property. For example, double-layer materials are used to achieve rapid moisture distribution or moisture dissipation into the next layer [5]. For all materials, but especially for barrier layers such as encasings and mattress protectors, sufficient breathability of the material must be ensured to prevent moisture build-up [6]. The water-vapor transmission resistance is determined in accordance with DIN EN ISO 11092 [7].

**4. Recommendation**

*The patient bed should meet ergonomic requirements to ensure hygiene and staff protection.*

**Recommendation degree:** ↑

**Strength of consensus:** >95%

The patient bed should allow technically assisted adjustment of the bed position, be easy to move and easy to reprocess [8].

**5. Recommendation**

*The bed frame and additional parts fitted to the bed should allow cleaning and disinfection.*

**Recommendation degree:** ↑↑

**Strength of consensus:** >95%

Electrically and mechanically operated hospital beds are class 1 non-critical medical devices (MD), which means that the design of all components, including movable and additional parts, must allow cleaning and disinfection. The surfaces of the bed frame must be smooth, easy to dry, and resistant to disinfection procedures. Components that do not allow this, e.g., electric motors that are not protected against water, electronic control consoles, etc., should not be used. Components in which liquid residues can remain are not permitted. Hollow bodies used in the design must be reliably sealed so that they are liquid-tight. 

A considerable amount of time is required to professionally reprocess beds fitted with butterfly screws. The four movable modules must be removed from their mountings (this requires great force) so that they can be cleaned from the underside. There are holes in the sides of the middle elements where, for example, the bed remote control can be hung. Dirt collects here and is difficult to remove. The movable modules have so many corners, edges and crevices that the bed has to be moved up and down to reach these areas for cleaning. Blood etc. collects in the grooves of the side rails, some of which have to be pushed into each other, and this can no longer be removed. The upper ends of the side rails are made of rough rubber material and do not form a smooth, closed surface that cannot be professionally cleaned and disinfected.

**6. Recommendation**

*To protect the mattress from contamination, wipe-disinfectable, liquid- and pathogen-proof mattress covers (encasings) should be used.*

**Recommendation degree:** ↑

**Strength of consensus:** >95%

*Staphylococcus (S.) aureus*, methicillin-resistant *S. aureus, Pseudomonas (P.) aeruginosa, Enterococcus (E.) faecalis*, coliform bacteria, Salmonella spp., vancomycin-resistant enterococci (VRE) and *Clostridioides* *(C.) difficile* [9], [10], [11], [12], [13], [14], [15], [16], [17], [18], [19] have been detected on mattresses. Contaminated mattresses have been the source of outbreaks of *P. aeruginosa* [20], gentamicin-resistant *P. aeruginosa* [21], *Acinetobacter (A.) calcoaceticus*, multidrug-resistant *A. baumannii* [22], [23], [24], VRE [25], MRSA [26], [27] and OXA-48-producing *K. pneumoniae* [28]. In mattresses made of polyurethane foam in children’s beds, not only the persistence but also the proliferation of *S. aureus* was detected, apparently caused by the accumulation of water-soluble substances including proteins in the polyurethane foam [29].

Encasings protect the mattress from contamination. Encasings must be impermeable to bacteria, proven by testing under exposure to moisture and pressure [30], [31], washable, wipe-disinfectable, and impermeable to liquids. Testing of the barrier function showed that even the penetration of radionuclides was prevented [32]. The encasing should at least enclose the sleeping and side surfaces [32], [33]. Easy-to-open full covers are advantageous. Encasings ensure that the mattress only needs to be reprocessed in the event of visible contamination, e.g., after damage to the encasing. Upon discharge, the covers are subjected to disinfectant wiping with the same cloth as the bed frame. 

When using wipe-disinfectable, pathogen-proof drapes with reprocessing after each patient change (washing with hot water with added surfactant and chlorine bleach), the rate of *C. difficile*-associated diarrhea (CDI) was reduced by 50% [34]. The preventive effect was especially high in combination with an antibiotic-stewardship strategy (reduction of 59% [35]). As the removal of encasings with subsequent washing is likely to lead to increased wear, personnel costs, and resource consumption, wipe disinfection with a sporocidal surface disinfectant is preferable.

Because sheets do not provide sufficient protection against contamination of the mattress [36], [37], [38], the CDC/HICPAC [39] recommend cleaning and disinfecting mattresses after each discharge if encasings are not used. As early as 2005, 96% of the mattresses at Greifswald University Hospital were fitted with encasings [40].

Another advantage of encasings, in contrast to cotton covers, is that there is no increase in the mite antigen concentration under the cover. The colonization with moulds and bacteria was also significantly lower under the encasing [41], [42]. The development and persistence of house dust mites and house dust mite allergens is an important factor for people with immune hypersensitivity. In cases where a person is immunocompromised, has an underlying infection or other predisposing factors such as asthma that make them susceptible to infectious diseases, allergen accumulation in the patient environment can have a strong impact on health [43], [44].

**7. Recommendation**

*It should be possible to prepare the mattress separately from the bed frame.*

**Recommendation degree:** ↑

**Strength of consensus:** >95%

For special situations, it should be possible to reprocess the mattress separately from the bed frame, e.g., using steam disinfection. This is the case, for example, if the encasing proves to be damaged when the patient is changed and the patient had an infection with a carbapenem resistant Gram-negative organism, a norovirus infection, a CDI or an infection with another critical pathogen. In cases where separate reprocessing is not possible, the mattress must be disposed of.

**8. Recommendation**

*For pillows and blankets, either pathogen-proof covers (encasings) or materials that can be reprocessed in a disinfection washing process should be used.*

**Recommendation degree:** ↑↑

**Strength of consensus:** >95%

Unless encasings are used, bed and pillow fillings should be reprocessable using the disinfection washing process, i.e., fillings with plant fibers, animal hair, down, thermolabile or poorly drying synthetic materials are therefore unsuitable [45]. At the same time, it must be ensured as a requirement for the laundry that fillings are completely dried after the washing process. 

Comforters and fitted sheets are also available as a unit (e.g., “All in One”) and can be washed at high temperatures. This saves time and has the advantage that every patient receives a fresh cover and comforter.

### 2. Bed reprocessing after discharge

**9. Recommendation**

*Every patient in the hospital should receive a prepared, clean bed that poses no risk of infection.*

**Recommendation degree:** ↑↑

**Strength of consensus:** >95%

Every patient has the right to a clean, disinfected bed covered with fresh linen [46], [47].

Used hospital beds are contaminated with the patient’s flora (bacteria, fungi, viruses, parasites) and can become a source of nosocomial infections (NI) if they are not reprocessed appropriately after reoccupancy [47], [48]. This applies to bed linen, pillows, comforters, mattresses, grab rails and other parts of the bed within easy reach, such as electrical switches, bells, and electronic control consoles. Studies on the contamination of patient beds and outbreaks, based on contamination of the bed, mainly date from the period 1990 to 2006; the bed has since been recognized as a potential reservoir of pathogens and has lost importance as a reservoir of NI due to disinfecting reprocessing.

Contamination of the bed is especially critical in the event of colonization or infection with multi-drug resistant organisms (MDRO) and pathogens with outbreak potential. Frequently touched surfaces on and near the bed, e.g., bed controls, call buttons and bedside tables, represent an important MDRO reservoir [49], [50]. Pathogens are released to a greater extent in diseases such as pneumonia with productive cough, urinary tract infections with incontinence, gastroenteritis, and cystic fibrosis [51], [52], [53], [54]. When assessing the risk of infection, it must be considered that hospital patients are more susceptible to infection than, for instance, healthy hotel guests, due to their illness, the presence of devices, and lowered immune defenses.

If a patient is colonized or infected with MDRO, and the following patient acquires the same pathogen, the pathogen is likely to originate primarily from the previous patient’s bed or immediate environment, because not all contaminated surfaces were reached by the final disinfection after discharge. In detail, however, the source of contamination remains unclear. Repeatedly, after discharge of patients who were colonized or infected with MDRO, subsequently admitted patients acquired the same pathogen due to deficiencies in the final disinfection [55], [56], [57], [58], [59], [60], [61], [62], [63]. This applied to both outbreaks and sporadic infections [61]. An analysis of 33,494 patients revealed an increased risk of acquiring MDRO when intensive care beds are occupied [64]. It is not clear from the analysis whether the bed also plays a role in this. However, the bed frame was more frequently contaminated with MDRO in infected ICU patients than in patients who were only colonized [65]. In any case, it is necessary to carefully include the area close to the patient, with the bed as the key target in the final disinfection [47]. 

In conclusion, unlike hotel beds, hospital beds should not only be cleaned but also disinfected to prevent colonization or infection by pathogens and their spread. Clear reprocessing instructions must be implemented for this [47], [48], [54]. The success of the reprocessing procedure should be checked and documented.

**10. Recommendation**

*Before each new occupancy, the handle-contact surfaces of the patient bed, movement aids, positioning aids, the surface and handle-contact surfaces of the bedside cab* *i* *net, the patient telephone and other control panels, as well as the closet, should be disinfected and cleaned.*

**Recommendation degree:** ↑↑

**Strength of consensus: **>95%

**Figure 1:**
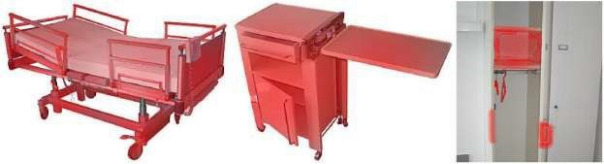
Surfaces to be disinfected before re-occupying the bed are marked in red

The necessity of bed reprocessing is demonstrated by the detection of representatives of the bedridden patient’s microflora on the bed frame as well as on the mattress and in the area close to the bed. Potentially pathogenic pathogens, often characterized by antibiotic resistance, were released in the area close to the patient, including the bed [66]. Catalano et al. [67] isolated an epidemic strain of *A. baumannii* from the bedrail during a four-month outbreak in an intensive care unit, concluding that although the bedrail per se does not explain the transmission, it shows that dry vectors can serve as secondary reservoirs. MRSA [15], [17], [68], *C. difficile* [69], [70], vancomycin-resistant enterococci (VRE) [19], [71] and *Candida auris* [72] were also detected on bedrails. The following species were detected at the following sites: Bacillus spp*.*, coagulase-negative staphylococci (CoNS), *Serratia plymuthica*,* Citrobacter (C.) koseri*,* C. braakii*,* K. pneumoniae*, and *A. baumannii* found on the bedside table; at the head of the bed coagulase-negative staphylococci CoNS; *Serratia odorifera*, Pantoea spp., Cronobacter spp*.*, and Mucor spp*.*; on the side rail of the bed CoNS, Bacillus spp., *Raoultella ornithinolytica*, Shigella spp*.*, *Enterobacter cloacae*, Pantoea spp*.*, *Serratia odorifera*, and *Haemophilus (H.) influenzae*; on the foot of the bed CoNS, Bacillus spp*.*, *Enterobacter amnigenus*, *Pseudomonas* *luteola* and *Moellerella wisconsensis*; and on the table above the bed CoNS, *K. pneumoniae*, *Enterobacter cloacae*, Bacillus spp*.*, *H. parainfluenzae* and Shigella spp*.* [73]. The MRSA infection rate was reduced by 96% (p <0.0001) by implementing a hospital-wide protocol for the disinfectant cleaning of surfaces close to patients [74]. In Figure 1 (Fig. 1), the surfaces to be included in disinfectant cleaning are marked in red.

**11. Recommendation**

*After the patient has been discharged, if mattress encasings are used, they should be disinfected by wiping and checked for damage.*

**Recommendation degree: **↑↑

**Strength of consensus:** >95%

Encasings can be disinfected by wiping with the same cloth as the bed frame. During wipe disinfection of the encasings, a visual check should be carried out to determine whether the cover is defective, soaked, or soiled [75]. If this is the case, it is removed and disposed of or reprocessed. Otherwise, the mattress may be contaminated, putting the patient at risk. The necessity of the inspection is demonstrated by a study in which 32.5% of 2,561 mattresses showed damage to the encasings. A distinction was made between 4 degrees of damage: no visible damage, minor damage that can be sealed with an approved repair patch, damage that cannot be repaired and should be replaced if possible, and severe damage with liquid flowing into the mattress and immediate replacement of the mattress [76]. Depending on the duration of bed rest and risk assessment for the release of critical pathogens, encasings should be opened for visual inspection of the mattress to determine whether liquid has penetrated. If this is the case, the encasing and the mattress should be replaced.

In the majority of bed-associated nosocomial outbreaks, damaged mattresses were the cause [77]. Bradbury et al. [78] reported that, upon visual inspection after a near-miss patient incident, 177 of 656 (27%) hospital-bed mattresses proved to be damaged and contaminated. From January 2011 to January 2016, the US Food and Drug Administration (FDA) [75] received more than 700 reports of medicalmattress covers that failed to prevent blood and body fluids from entering the mattress. In response, the FDA issued a safety communication that provides recommendations to healthcare providers, healthcare facility staff, and caregivers, for the inspection, maintenance, replacement, and removal of mattresses in healthcare facilities [74]. The FDA [75] recommends that bed-managing hospitals develop a mattress inspection plan and immediately replace any mattress cover showing signs of damage or wear. Following the FDA’s communications, the Joint Commission published guidance in 2018 [79] specifically stating that healthcare facilities should avoid “tears or holes in upholstery or mattresses”, and “patch any holes or tears with an approved product (not tape) that can be cleaned and disinfected. It is therefore advisable to have a risk-adapted inspection plan for the beds.”

**12. Recommendation**

*For disinfecting cleaning of the bed frame, including accessories and encasings, surface disinfectants with manufacturer-independent, expert-assured effectiveness should be used.*

**Recommendation degree:** ↑↑

**Strength of consensus: **>95%

The disinfectants must meet the following requirements for use in infection-sensitive areas that go beyond the biocidal product approval [80]

- The disinfectant must have been tested singly.
- The reproducibility of the results must be confirmed by two independent laboratories. Specifications for replication of the efficacy test must be observed.
- The bactericidal and levurocidal efficacy and any additional tuberculocidal, mycobactericidal, fungicidal, sporicidal and/or virus-inactivating efficacy required as a result of the risk assessment must be documented by two independent test reports and associated expert opinions.
- The test reports must fulfill the requirements for the test method according to the current state of scientific knowledge, consider the actual application form or technique, and have been prepared by accredited laboratories independent of the manufacturer.
- The test reports and expert opinions with recommendations for use must be scientifically evaluated by an independent expert commission for conformity with standardized requirements.

These requirements are met by products and procedures that are included in the disinfectant list of the German Association for Applied Hygiene (VAH) [81], the Austrian Society of Hygiene and Microbiology (ÖGHMP) or the Robert Koch Institute (RKI) in Berlin [82]. Products or procedures from these lists guarantee a high level of safety. Therefore, only preparations included in these lists should be used [80].

**13. Recommendation**

*If there is any indication of contamination with non-enveloped viruses, mycobacteria, bacterial spores or fungi, surface disinfectants with the appropriate spectrum of activity should be used.*

**Recommendation degree:** ↑↑

**Strength of consensus:** >95%

International consensus exists on the need for disinfectant surface cleaning in line with indications [47]. This applies especially to frequently touched surfaces close to the patient, such as the bed and its surroundings [83]. The CDC recommends, for example, that patients infected with *Candida auris* should be placed in isolation with daily disinfectant cleaning of surfaces close to the patient and final disinfection after discharge [72].

Both reviews and guidelines on preventing the transmission of, e.g., *C. difficile*, MRSA and noroviruses recommend controlled disinfectant surface cleaning as part of the prevention strategy [84], [85], [86], [87], [88], [89], [90], [91], [92], [93], [94], [95], [96], [97], [98], [99], [100], [101], [102], [103]. This is even more pronounced in outbreak situations [104], [105]. 

With the exception of mycobacteria, bacterial spores, fungi or non-enveloped viruses, all vegetative bacterial species (bactericidal) and yeasts (levurocidal) are killed by VAH-listed surface disinfectants [80]. Disinfectants with an additional, appropriately declared spectrum of activity may be characterized by higher cytotoxicity (depending on the active substance and/or the application concentration) [106], which may increase the risk of side effects. Therefore, surface disinfectants that include the entire spectrum of activity should not always be used. Instead, the choice should be made based on a careful risk-benefit assessment. In case of infection with *Mycobacterium tuberculosis* or atypical mycobacteria, disinfectants with the “tuberculocidal” or “mycobactericidal” spectrum of activity should be used, especially for final disinfection and in outbreak situations [3]; the relevant information can be found in the VAH list [81]. After contamination with bacterial spores (e.g., infection with *C. difficile*), the spectrum of activity should be sporicidal, for non-enveloped lipophilic viruses (e.g., infection by noroviruses, rota viruses or adenoviruses) the spectrum of activity should be limited to virucidal Plus, for non-enveloped hydrophilic viruses (e.g., infection by HEV, HAV, parvoviruses, boca viruses, picornaviruses) it should be virucidal, and for molds (e.g., pulmonary aspergillosis) it should be fungicidal [47], [107]. In the rare case of disinfection ordered by the authorities in accordance with Section 18 (1) sentence 1 no. 1 of the Infection Protection Act, surface disinfectants from the Robert Koch Institute Berlin disinfectant list should be used [82].

For reprocessing, the requirements for the general hygienic reprocessing of medical devices in accordance with Section 4 (1) of the Medical Devices Operator Ordinance (MPBetreibV) must be met, as well as the requirements for special hygienic reprocessing in accordance with Section 4 (2) MPBetreibV [108]. If country-specific regulations on bed reprocessing exist, these must be complied with [109].

**14. Recommendation**

*The bed linen (comforter cover, pillowcase and sheets) should be disinfected before each new occupancy.*

**Recommendation degree:** ↑↑

**Strength of consensus: **>95%

This is necessary to protect patients from infection. In addition to the risk of infection from contaminated laundry, microbial proliferation on textiles can caused unpleasant odors and skin irritation, and reduces the tear resistance of textiles [107], [108].

**15. Recommendation**

*Reprocessed bed linen should be visually and functionally flawless and treated in such a way that patients are not endangered by detergent residues at irritating concentrations.*

**Recommendation degree:** ↑↑

**Strength of consensus:** >95%

As detergent residues can cause skin irritation [110], [111], [112], [113], it is advisable to ask the laundry whether their quality assurance system excludes excessive detergent residues, e.g., by using rinse test fabrics. The following values are recommended as guides in the rinse test fabric: pH value 4.3–8.3; organic incrustation <1.0%; inorganic incrustation <1.0%; anionic surfactants <200 µg/g; nitrogen surfactants <450 µg/g for PES/CO (Polyester/Baumwolle) blends, and <600 µg/g for cotton [114]. Detergent stains can be an indication of excessive residues. Laundries should ensure that the reprocessed bedding no longer has any residual alkalinity by acidifying the rinsing bath accordingly and using sufficient rinsing baths. If a skin-irritating effect is suspected, it is recommended that the reprocessing process in the reprocessing laundry is checked by a representative of the hygiene staff. In one such suspected case, the pronounced cytotoxicity of the reprocessed laundry was demonstrated in a cell culture test. After checking the procedures in the laundry and changing the reprocessing process (acidification in the rinsing bath and subsequent rinsing baths), the cytotoxicity was no longer present (Kramer, unpublished). Craemer and Humphries [54] described problems resulting from inadequate cleaning of hospital beds. They argued that bed linen should be reprocessed once a week if patients are at specific risk of infection. Optimal bed linen was described as that which can be easily washed and dried and has the lowest potential to harbor microorganisms. In addition, it was reiterated that pillows and mattresses deserve the most attention due to their proximity to the patient. The correct maintenance of positioning presses and trolleys as part of the reprocessing process was highlighted as an area that should also be considered as part of the prevention strategy.

**16. Recommendation**

*Sheets and bed linen should be completely removed after each patient change and collected directly in sufficiently resistant and clearly labeled laundry bags without intermediate storage.*

**Recommendation degree: **↑↑

**Strength of consensus:** >95%

After discharge, the bed linen is completely removed and collected without intermediate storage. This is to ensure that pathogens adhering to the bed linen are not spread further. Coordination between the work areas in which the linen is produced and the laundry is required for correct collection (e.g., wet laundry transport with additional liquid-tight bag) and labeling [40].

**17. Recommendation**

*Sheets and bed covers should be reprocessed after each patient change using a validated disinfecting washing process.*

**Recommendation degree:** ↑↑

**Strength of consensus: **>95%

On dry textiles, pathogens retain their ability to multiply for days to weeks, e.g., *E. faecium* up to 51 d, *E. faecalis* >90 d, VRE >80 d, MRSA >206 d, *Streptococcus pyogenes* >206 d,* A. baumannii *>60 d, *Escherichia coli* >206 d, *K. pneumoniae* >56 d, *P. aeruginosa* >56 d, *Stenotrophomonas maltophilia* >52 d, *A. calcoaceticus anitratus* >25 d, *A. calcoaceticus* *lwoffii* 7 d, *A. niger* >30 d, *C. albicans* 120 d, *Cryptococcus neoformans* >30 d, hepatitis A virus (on paper) >60 d, hepatitis E virus (on stainless steel) >28 d, rotavirus >7 d, influenza A virus 14 d, papillomavirus <7 d, hepatitis B virus >14 d [115]. SARS-CoV-2 was infectious on clothing at 20°C for up to 14 d [116]. Scabies mites persist on bed linen for 24–36 h [117]. The role of bed linen as a source of contamination for scabies became evident, for example, when bed linen was returned from the laundry that had not been properly reprocessed, resulting in a nosocomial outbreak of scabies [118]. *S. aureus*, *E. faecium*, *P. aeruginosa* and *Enterobacter* *aerogenes* can withstand temperatures of 60°C in standard washing procedures [119]. Therefore, bed linen must be reprocessed in a validated disinfecting washing process to safely inactivate adhering pathogens. For chemothermal disinfection-washing procedures, the concentration of the respective detergent and disinfectant, the solvent ratio during the disinfection phase, the temperature and the temperature holding time as well as the effective range, are specified [81], [82]. Laundry disinfection processes are predominantly peracetic acid-based processes with temperatures between 60 and 70°C; however, 40°C processes are also available [81], [82].

As expected, all samples of used bed linen were microbially contaminated with a mean load of 23 colony-forming units (CFU)/25 cm^2^ (range 1–191 CFU). 57% of the species were potentially pathogenic [120], which poses a particular risk for patients with immune deficiencies.

**18. Recommendation**

*Pillows and comforter cores (inlets) should only be disinfected on an ad hoc basis and in accordance with a risk assessment at specified intervals.*

**Recommendation degree:** ↑↑

**Strength of consensus: **>95%

Soiling, moisture penetration, sweat marks, odor, defects and contamination with critical pathogens can be reasons for a necessary change. Defective pillow and comforter cores must be repaired or sorted out and replaced. Storage capacity is required for this purpose.

The decision to carry out disinfecting reprocessing on an ad hoc basis should be based on a risk assessment by the ward management and the hygiene team. In the case of known colonization or infection with MDRO or with pathogens of transmissible infections, disinfecting reprocessing is indicated after discharge in risk areas (e.g., intensive care, isolation unit, transplant units, patients with chronic wounds, catheters or incontinence problems). The following findings justify the need for the risk assessment. Acinetobacter spp. [121], [122], Aspergillus spp., *Aureobasidium* *pullulans* and *Rhodotorula* *mucilaginosa* [123] as well as MRSA [13] were detected on pillows. In a study by Lange et al. [124], 38% of hospital pillows were colonized with MRSA and coliform bacteria. After cutting open nominally fluid-tight pillows with seams from a burn unit, many were visibly contaminated with body fluids [125]. Mottar et al. [126] observed striking discrepancies in the weight of pillows in a burn center. The pillows were found to be a source of leakage, and several pathogens were isolated from inside the pillows that correlated with patient infections. Lippmann et al. [127] searched for reservoirs of infection to explain a large outbreak of 4 MRGN *K. pneumoniae* in Germany and found that positioning pillows were internally contaminated, with pathogen persistence for at least 6 months. Carbapenem-resistant Enterobacteriaceae were detectable on the bed frame, pillow and mattress pad [128]. No test results on microbial contamination of comforters were available in the literature.

If there is no indication of infection risk, reprocessing can be omitted after the patient has been discharged, and reduced to the defined reprocessing frequency. However, there is no evidence upon which to base a standardized interval of regular reprocessing. As the frequency is influenced by the frequency of occupancy changes and the patient clientele, it should be determined in consultation between the hygiene specialist and the ward management. For pillows, a quarterly cycle appears to be a sensible guideline. As fitted blankets are less sweaty than pillows, a six-month cycle may be sufficient. Where encasings are used, these can be wipe-disinfected; otherwise, the comforter must be reprocessed by disinfection washing. 

So-called barrier cushions made of vinyl offer the highest level of safety. They differ from standard encasings in that the seams are not sewn but welded together with high frequency radio waves to achieve a seal. The lack of seam holes prevents contaminated air from penetrating via the seams. Instead, the air flows in and out of the cushion through a watertight filter that prevents the penetration of bacteria, fungi and viruses down to a size of 25 nm [129]. The pillowcase is disinfection-cleaned before reuse. When comparing 100 of these covers with 100 new nominally occlusive pillows with stitched seams, 60% of the inside of the standard covers were contaminated after 3 months of use, while none of the barrier covers were contaminated. This led to a company-wide introduction of the barrier cover and an associated reduction in infections caused by MRSA and *C. difficile* [130].

**19. Recommendation**

*If final disinfection is required, the bed and the area around the bed should be included in the disinfection cleaning process.*

**Recommendation degree:** ↑↑

**Strength of consensus:** >95%

In case of deficiencies in the final disinfection after discharge of patients with problematic pathogens, infection of the next patient with the pathogen was observed in a number of studies [47]. Further information on indications for final disinfection can be found in the recommendation of the Commission for Hospital Hygiene and Infection Prevention (KRINKO) concerning hygiene requirements for cleaning and disinfection of surfaces [47].

**20. Recommendation**

*For patients with Creutzfeldt-Jakob disease (CJD), the reprocessing procedure should be coordinated with the responsible hospital hygienist.*

**Recommendation degree:** ↑↑

**Strength of consensus:** >95%

Due to the high resistance of prions, the procedure should be decided upon depending on the risk [131], [132].

**21. Recommendation**

*Reprocessed beds should be clearly labeled as “reprocessed”.*

**Recommendation degree: **↑↑

**Strength of consensus:** >95%

Otherwise, there is a possibility that beds that have not been prepared will be occupied by the next patient.

**22. Recommendation**

*If the patient will just be lying down for a few hours, stretchers or couches with washable and disinfectable surfaces can be used instead of a bed.*

**Recommendation degree:** ↔

**Strength of consensus: **>95%

If the length of stay is only a few hours, e.g. for outpatients, day clinics and dialysis patients, we recommend placing the patient on surfaces that can be washed and disinfected without sheets, comforters and pillows for reasons of sustainability. The nature of the bed must offer sufficient comfort. If the bed surface is provided with a fresh cover for each patient, the bed should only be wiped clean and disinfected after contamination, including all contact surfaces. Although no studies are available on this, couches/stretchers should be completely disinfected at approximately weekly intervals. 

### 3. Bed hygiene during the patient’s stay

**23. Recommendation**

*The contact surfaces close to the patient (e.g., on the bed, bedside cabinet, control elements) should be disinfected once a day.*

**Recommendation degree: **↑

**Strength of consensus:** >95%

Since adherence to hand antisepsis varies between 9.1% and 85.2% depending on the type of ward, geographical region and team leadership [133], [134], daily disinfection of frequently touched surfaces close to patients reduces the risk of spreading nosocomial infections [47].

**24. Recommendation**

*In the case of visible contamination, the bed frame should be cleaned and disinfected promptly. Depending on the extent of the contamination, a two-stage proces* *s for c* *lean* *ing and disinfection can be considered.*

**Recommendation degree:** ↑↑

**Strength of consensus:** >95%

As a basic hygiene measure, contamination with potentially pathogen-containing material, e.g., blood, secretions or excretions, should be promptly removed mechanically first by cleaning (without disinfectants due to protein fixation). Only afterward should disinfectant surface cleaning or surface disinfection be carried out (two-stage procedure) [47]. Contaminated bed linen must be changed promptly.

**25. Recommendation**

*Bed linen can be changed weekly for each patient.*

**Recommendation degree:** ↔

**Strength of consensus: **>95%

Studies on the necessary frequency of linen changes are not available. Changing the bed linen at least once a week has proven to be effective [46], [54].

**26. Recommendation**

*Visibly soiled or sweaty bed linen should be changed and reprocessed promptly.*

**Recommendation degree:** ↑

**Strength of consensus:** >95%

Independent of the possible risk of contamination due to proliferation of potential pathogens in a moist environment, reprocessing is necessary from an esthetic point of view. As contamination cannot be eliminated by disinfection, there is no alternative to replacement.

**27. Recommendation**

*Post-intervention, the patient can be returned to bed without changing bed linen.*

**Recommendation degree:** ↔

**Strength of consensus: **>95%

If the patient was only in the hospital for a short time for perioperative preparation and the bed is not contaminated, it makes sense to continue using the bed for the same patient without changing the linen, as a contribution to sustainability.

**28. Recommendation**

*In the case of antiseptic decolonization of MRSA, sheets and comforter covers should be changed daily during de* *colonization. Mattress and pillowcases should be subjected to wipe disinfection prior to daily re-covering.*

**Recommendation degree:** ↑↑

**Strength of consensus: **>95%

It is common practice to change the patient’s clothing and washing utensils daily during antisepsis in addition to changing the bed linen [135], [136]. Otherwise, recolonization of the patient will occur.

For intensive care patients with devices, the need to change linen should be determined by the ward management together with the hygiene team following a risk assessment.

### 4. Personal protection when handling beds

**29. Recommendation**

*Personal protective measures should be observed when removing and collecting used laundry that is contam* *i* *n* *a* *t* *ed with body fluids and excretions and for patients in contact or physical isolation.*

**Recommendation degree: **↑↑

**Strength of consensus: **>95%

According to TRBA 250 [137], these are activities of protection level 2. Therefore, protective gowns, gloves, and, depending on the pathogen, mouth, nose, and hair protection should be worn both for self-protection of the team and to prevent the further spread of nosocomial infections in the case of airborne or respiratory pathogens. Information on handling laundry from areas with an increased risk of infection can also be found in DGUV Information 203-084 [138].

**30. Recommendation**

*Before the regular technical inspection, the bed shou* *ld b* *e thoroughly cleaned and disinfected.*

**Recommendation degree:** ↑↑

**Strength of consensus:** >95%

The aim of the technical inspection is to ensure functionality as well as user and patient safety. Beds equipped with electrical systems are tested and maintained by certified customer service technicians [139], [140]. The inspection is documented and the beds are provided with a test seal. Before the inspection, the bed should be thoroughly disinfected, as the underside of the bed frame may have been soiled and contaminated by dust with adhering pathogens and by splashes during floor cleaning.

**31. Recommendation**

*After contamination of the bed linen with hazardous drugs, occupational health and safety measures should be applied to prevent any risk to the treating/caregiver staff.*

**Recommendation degree:** ↑↑

**Strength of consensus:** >95%

The following principles must be observed [141], [142]:

- Laundry contaminated with hazardous drugs (HD), e.g., with cytostatic drugs, should only be touched with gloved hands, and the gloves must be certified for handling cytostatic drugs.
- If laundry contaminated with HD can come into contact with clothing during handling, e.g., when stripping the bed, a gown should be worn in addition to the gloves.

According to the guidelines of the Occupational Safety and Health Administration [143], laundry contaminated with HA-contaminated excretions should be treated according to the standard for bloodborne pathogens. In addition to wearing personal protective equipment, this includes

- handling laundry as little as possible and not sorting or rinsing,
- packing laundry in the place where it was used,
- packing contaminated laundry in a labeled or color-coded bag so that staff can identify the nature of the contents,
- use a bag that prevents leakage if the contaminated laundry is so wet that seepage or leakage is possible during transportation, consider double bagging if necessary.

According to OSHA [144], the contents of laundry bags with HD-contaminated laundry should be prewashed and only then should the laundry be added to other laundry for a second wash cycle. If such laundry is present, the reprocessing must be coordinated with the contracted laundry.

**32. Recommendation**

*Reprocessed, freshly made beds can be covered until they are used again.*

**Recommendation degree:** ↔

**Strength of consensus: **>95%

After reprocessing, the bed is covered with clean bed linen and, if necessary, covered to protect it from soiling and dust if it is not used for a longer period of time. Recyclable plastic sheeting is suitable for covering the bed.

### 5. Organization of bed reprocessing

**33. Recommendation**

*Bed reprocessing can be organized decentrally or* *cent* *rally.*

**Recommendation degree:** ↔

**Strength of consensus:** >95%

At the beginning of the 1990s, centralized bed reprocessing was still preferred on the assumption that decentralized reprocessing resulted in uncontrollable risks of cross-infection, that there was no storage space for reprocessed beds on the ward, and that numerous errors were observed in decentralized reprocessing. When reprocessing in the patient's room, the lack of separation of clean and unclean activities and an overall unsystematic approach were observed [145]. 

Due to the significantly lower costs and the technical complexity of modern hospital beds, bed reprocessing is now almost exclusively carried out decentrally, even if there is still a central bed reprocessing unit. With identical reprocessing results, the total costs for centralized manual and centralized automated reprocessing are 2.9 and 4.5 times higher, respectively, compared to decentralized bed reprocessing. In addition, repairs to the bed frame are more frequent with centralized reprocessing, and the wear and tear on mattresses increases [146]. Finally, there is a risk of spreading pathogens when transporting beds to be reprocessed to the bed center. The preference for decentralized bed reprocessing is also supported by the fact that patients and visitors in the hospital are not confronted with bed transports and the load on the freight elevator caused by bed transports is eliminated. In the case of centralized reprocessing, it is desirable to equip the bed center with a bed lifting system to facilitate cleaning and maintenance work on the patient beds [8]. 

In the case of decentralized reprocessing, the manual reprocessing of the patient bed should be carried out on the ward level in a separate room to avoid disturbing other patients. In multi-bed rooms, another reason for separate reprocessing is that no nursing or medical activities can be carried out on other patients in the patient room during reprocessing. When planning, it is recommendable to provide a bed reprocessing room that can be conveniently used by one or more wards. The room should enable the functional separation of work processes into unclean and clean, be equipped with a dosing device for preparing disinfectant solutions, and ideally contain a bed lifting system [8].

In single-bed rooms, from a hygiene point of view, reprocessing can also be carried out in the room, because bed and patient have been in the room up to this time and dust and pathogens are not fundamentally different from those generated during daily bed-making. Even given cohort isolation, e.g., in a twin room, bed reprocessing including disinfection of other surfaces close to the patient can be carried out after discharge from the isolation unit. Once isolation has been lifted, the patient room must undergo final disinfection before being reoccupied. 

If no reprocessing room is available on the ward, reprocessing can be carried out in the occupied multi-bed room as temporary solution. This procedure causes no additional infection load; however, it is a nuisance. In any case, good room ventilation must be ensured during disinfecting reprocessing. The hygiene team should define the framework conditions for this temporary solution (choice of disinfectant, ventilation, integration into the ward routine).

**34. Recommendation**

*Ward corridors should not be used to reprocess beds.*

**Recommendation degree:** ↑

**Strength of consensus:** >95%

There is a risk of cross-infection. At the same time, corridor use is obstructed, escape and rescue routes can be blocked and visitors are left with a poor impression. 

### 6. Quality assurance of bed hygiene

**35. Recommendation**

*In both decentralized and centralized bed reprocessing, all sub-steps of reprocessing should be determined by the persons or companies responsible for reprocessing, in coordination with hospital hygiene. All reprocessing should be carried out by trained personnel and personnel protection should be ensured.*

**Recommendation degree:** ↑↑

**Strength of consensus:** >95%

According to the Medical Devices Operator Ordinance [108], the reprocessing of MD must be specified in all sub-steps. Proper reprocessing is assumed if the joint recommendation of the KRINKO and the Federal Institute for Drugs and Medical Devices (BfArM) [147] on the “Hygiene requirements for the reprocessing of medical devices” is observed.

For both centralized and decentralized manual reprocessing, each sub-step must be defined in detail (cleaning, drying, disinfection, functional testing) and the resources must be defined and documented as work instructions (e.g., in the hygiene plan or as standard operating procedure). 

A random visual and microbial inspection by hygiene staff of freshly reprocessed beds for residual soiling and microbial contamination is recommended every six months [148].

Decentralized bed reprocessing can be assigned to the collection and delivery service or the cleaning service [146]. Reprocessing by the cleaning service is the most cost-effective option [146]. A prerequisite is the training of staff, e.g., as trained bed reprocessing teams [40]. This does not exclude the possibility that nursing staff can also perform this activity outside of core working hours, for example. In any case, the type of service selected for reprocessing is responsible for the hygienic transfer. The hygiene staff are responsible for the technical instruction, monitoring and control of the reprocessing quality. Further training for state-certified disinfectors is recommended every three years for the head of the reprocessing team.

**36. Recommendation**

*The preparation team should receive a daily report on the number of discharge beds that need to be prepared. Additionally, personnel should be available on-call to handle the preparation of unplanned discharge beds as needed.*

**Recommendation degree:** ↑

**Strength of consensus:** >95%

The preparation team should receive a daily report on the number of discharge beds that need to be prepared, broken down by category. This includes beds without infection risk, beds from high-risk areas, and beds occupied by patients with colonization or infection with MDRs or other critical pathogens. The criteria for the risk assessment should be specified in the hygiene plan to ensure appropriate risk classification. If there is uncertainty in classification, hygiene specialists can be consulted. This approach ensures a high quality of preparation. Additionally, personnel should be available on-call to handle the preparation of unplanned discharge beds as needed.

**37. Recommendation**

*To* *ensure the safety of preparation, a semi-annual procedural review should be conducted for decontamination systems used for beds, bedside tables, and mattresses.*

**Recommendation degree: **↑↑

**Strength of consensus:** >95%

The recommendations for semi-annual procedural reviews of washing systems using standardized test bodies contaminated with *E. faecium* in bovine serum albumin and mucin differ depending on the treatment of bed frames, bedside tables, and mattresses (process details in [149]). 

Additionally, compliance with physical procedural parameters should be verified using thermologgers.

**38. Recommendation**

*Before utilizing a hospital laundry service, it should be verified that a hygienically safe disinfection washing procedure is used.*

**Recommendation degree:** ↑↑

**Strength of consensus:** >95%

To ensure that hygienic requirements are met, quality assurance is needed from delivery through processing to contamination-safe storage [150], [151]. Laundries and textile service providers that process textiles from hospital settings must meet the requirements of the Robert Koch Institute [82] and establish a hygiene management system. For example, the RABC system (Risk Analysis and Biocontamination Control System) certifies processing safety based on a risk analysis according to DIN EN 14065 [152]. When contracting with the laundry, it should be ensured that confirmed auditing is in place. Quality assurance according to RAL-GZ 992/2 [153] also includes the requirements according to the RKI, including unannounced external monitoring as part of the hygiene management system, as well as monitoring the preservation of textile value [RAL-GZ 992]. The use of disinfection washing procedures with efficacy area AB [82] is recommended (A=killing of vegetative bacteria including mycobacteria and fungi, including fungal spores; B=inactivation of enveloped and non-enveloped viruses).

**39. Recommendation**

*When handling beds in the facility, damage to encasings, pillows, and mattress pads should be avoided and re* *por* *t* *ed if it occurs.*

**Recommendation degree: **↑↑

**Strength of consensus:** >95%

Care should be taken to maintain the integrity of encasings, pillows, and mattress pads to prevent the penetration of fluids and pathogens. This includes ensuring that no sharp objects, such as scissors, needles, syringes, scalpels, or other pointed items, are placed on the mattress surface [154]. Noticeable defects should be reported to the ward or department management so that corrective actions can be taken.

**40. Recommendation**

*In case of a nosocomial infection outbreak, microbio* *l* *o* *g* *i* *c* *al, virological, or parasitological testing of prepared beds may be appropriate upon recommendation by hygiene specialists.*

**Recommendation degree:** ↔

**Strength of consensus:** >95%

Since beds have been associated with outbreaks of nosocomial infections [20], [21], [22], [23], [24], [25], [26], [27], [28], [155], [156], [157], during outbreaks – especially involving pathogens with high persistence or, in the case of viruses, with high recoverability on textiles and simultaneously low infectious doses – beds of unclear origin can be included in the source investigation. Pathogens such as *C. difficile*, MRSA, *Acinetobacter* and Klebsiella spp., *P. aeruginosa*, noroviruses, and scabies mites (especially in cases of crusted scabies) should be considered [158]. One study reported colonization in newborns due to washed laundry contaminated with *B. cereus*. This contamination was apparently linked to a deficient washing process and high ambient temperatures in the ward [159]. 

As a microbiological quality indicator for prepared beds, microbial load can be determined using agar contact methods, for example, according to DIN 10113-2 [160] or DIN EN ISO 18593 [161].

## Notes

This guideline was originally published in German [162]. 

### Competing interests

The authors declare that they have no competing interests.

### Acknowledgement 

We would like to thank Dr. rer. Biol. Hum. Cathleen Muche-Borowski, MHP, representative of the Association of the Scientific Medical Societies in Germany, and the Institute for Medical Knowledge Management, for their support in the conflict of interest assessment and the formal consensus-finding process. 

### Funding 

The elaboration of the guideline was not financially supported. 

### Authorization by the participating societies 

The version of the guideline approved by the editorial committee was sent to the boards of the participating societies mentioned above before publication and was authorized and approved by all of them in toto without changes.

### Period of validity and updating procedure 

The guideline was last edited for content on 06/2024, and the validity period is set at 5 years. Comments on the update are welcome. 

Contact person is Prof. em. Dr. med. habil. Axel Kramer axel.kramer@med.uni-greifswald.de).

### Authors’ ORCIDs

Kramer Axel: https://orcid.org/0000-0003-4193-2149

Seifert J: https://orcid.org/0009-0000-8890-8934

Arvand M: https://orcid.org/0000-0002-7664-5150

Chaberny I: https://orcid.org/0000-0001-5859-3660

Müller W: https://orcid.org/0000-0003-4547-013X

Novotny A: https://orcid.org/0000-0002-8029-5958

Scheithauer S: https://orcid.org/0000-0003-0773-4739

Schulz-Schaeffer W: https://orcid.org/0000-0001-5886-2322

Sunderdiek U: https://orcid.org/0009-0002-0793-6146

### Guideline report

The guideline report can be viewed in Attachment 1.

## Supplementary Material

Guideline Report
